# Efficacy and Safety of Tofacitinib in the Management of Ankylosing Spondylitis: A Comprehensive Systematic Review

**DOI:** 10.7759/cureus.86556

**Published:** 2025-06-22

**Authors:** Mohammad Burhanuddin, Sikandar Abbas Chaudhry, Archana Dhami, Fatima Iqbal, Sundus Shafiq, Sami Ullah, Asma Khalid, Muhammad Wali Hassan, Muhammad Muaz Bhatti, Muhammad Uzair Siddique

**Affiliations:** 1 Medicine, Bhaskar Medical College, Hyderabad, IND; 2 Medicine, King Edward Medical University, Lahore, PAK; 3 Family Medicine, Avalon University School of Medicine, Willemstad, CUW; 4 Internal Medicine, Punjab Medical College, Faisalabad, PAK; 5 Medicine, Punjab Medical College, Faisalabad, PAK; 6 Internal Medicine, King Edward Medical University, Lahore, PAK

**Keywords:** ankylosing spondylitis, axial spondyloarthritis, disease activity, efficacy, jak inhibitor, mri inflammation, safety, tofacitinib

## Abstract

Ankylosing spondylitis (AS) is a chronic, immune-mediated inflammatory arthritis that can severely impair the quality of life. Although biologic therapies have significantly advanced disease management, a substantial number of patients continue to experience suboptimal treatment outcomes. This systematic review evaluated the efficacy and safety of tofacitinib, an oral Janus kinase (JAK) inhibitor, in the management of AS. We conducted a comprehensive search across multiple databases for studies published between 2015 and 2025, ultimately including 11 relevant studies (two randomized controlled trials (RCTs) and nine post-hoc or observational analyses). Evidence consistently demonstrated that tofacitinib 5 mg twice daily significantly improved clinical outcomes compared to placebo, with Assessment of Spondyloarthritis International Society (ASAS) response rates of approximately 80% in controlled trials. Tofacitinib demonstrated a rapid onset of action, with improvements in pain occurring within one month and benefits across multiple domains, including fatigue, morning stiffness, and quality of life. Magnetic resonance imaging (MRI) studies confirmed significant reductions in sacroiliac joint and spinal inflammation. The safety profile was consistent with that observed in other indications, with nasopharyngitis and upper respiratory infections being the most common adverse events. Limited cases of herpes zoster were reported, with no documented tuberculosis, malignancies, or thromboembolic events within the study periods. The review supports tofacitinib as an effective option for AS management, with efficacy maintained across various patient subgroups. However, longer-term studies and head-to-head comparisons with biologics are needed to establish its optimal position in treatment algorithms for AS.

## Introduction and background

Ankylosing spondylitis (AS) is a chronic, immune-mediated inflammatory arthritis primarily affecting the axial skeleton, particularly the sacroiliac joints and spine [1]. Classified within the broader spectrum of spondyloarthropathies, AS typically manifests in young adults and is characterized by chronic back pain, morning stiffness, progressive spinal rigidity, and, in advanced cases, significant functional impairment due to vertebral fusion. The prevalence of AS varies globally, with estimates ranging from 0.1% to 1.4%, depending on genetic predisposition and the frequency of the HLA-B27 allele, which is strongly associated with the disease [2]. The pathophysiology of AS is complex and multifactorial, involving genetic susceptibility, aberrant immune responses, and environmental triggers. Recent insights into disease mechanisms have emphasized the roles of pro-inflammatory cytokines such as tumor necrosis factor-alpha (TNF-α) and interleukin-17 (IL-17). The introduction of biologic therapies, particularly TNF inhibitors and IL-17 antagonists, has revolutionized AS treatment by providing significant symptomatic relief and disease control in many patients [3,4]. However, a substantial subset of patients either do not respond adequately or are intolerant to these agents, highlighting the need for alternative therapeutic options.

Tofacitinib is an oral small-molecule Janus kinase (JAK) inhibitor that modulates the JAK-STAT signaling pathway, a critical intracellular mechanism for transducing cytokine-mediated signals involved in immune regulation and inflammation. Initially approved for the treatment of rheumatoid arthritis (RA), psoriatic arthritis, and ulcerative colitis, tofacitinib has shown potential utility across various autoimmune diseases [5]. The rationale for exploring JAK inhibition in AS stems from the drug’s ability to target multiple inflammatory pathways simultaneously, potentially offering a broader and more robust anti-inflammatory effect than cytokine-specific biologics. Emerging clinical trials and observational studies have investigated the role of tofacitinib in managing AS. These studies have suggested promising results in terms of both symptom relief and suppression of inflammatory markers. Patients receiving tofacitinib have demonstrated improvements in clinical endpoints such as the Assessment of Spondyloarthritis International Society (ASAS) response criteria, Bath Ankylosing Spondylitis Disease Activity Index (BASDAI), and quality of life measures [6-8]. Moreover, the oral administration route of tofacitinib offers an advantage in terms of patient convenience and adherence compared to injectable biologics.

Despite these encouraging outcomes, safety concerns regarding tofacitinib have also emerged. Its immunosuppressive mechanism may predispose patients to infections, including opportunistic pathogens such as herpes zoster and tuberculosis [8]. Additionally, long-term safety data have raised concerns about potential risks of venous thromboembolism, malignancies, and cardiovascular events, especially in patients with preexisting risk factors. The balance between efficacy and safety remains a critical consideration in evaluating the role of tofacitinib for AS treatment. To date, there has been no comprehensive synthesis of the evidence regarding the safety and efficacy of tofacitinib specifically in AS. While individual studies provide valuable insights, a systematic review is warranted to integrate findings across multiple sources, assess the strength and consistency of the evidence, and identify areas requiring further research. Such an analysis is particularly relevant given the growing interest in JAK inhibitors and the evolving therapeutic landscape for axial spondyloarthritis.

This systematic review aims to evaluate the current body of evidence on the safety and efficacy of tofacitinib in the treatment of AS. By synthesizing data from clinical trials and observational studies, this review seeks to answer the following key questions: (1) effectiveness of tofacitinib in achieving clinical remission or low disease activity in patients with AS, (2) common and serious adverse events associated with its use in this population, and (3) risk-benefit profile of tofacitinib. The findings of this review will provide valuable guidance for clinicians considering tofacitinib as a treatment option for AS and will contribute to informed decision-making in clinical practice. Furthermore, it will identify gaps in knowledge and inform future research directions aimed at optimizing outcomes for patients living with this debilitating condition.

## Review

Materials and methods

Study Selection

This systematic review was conducted in accordance with the Preferred Reporting Items for Systematic Reviews and Meta-Analyses (PRISMA) guidelines. A comprehensive literature search was performed across multiple electronic databases, including PubMed, Scopus, Web of Science, Embase, and the Cochrane Library. The search aimed to identify relevant studies evaluating the safety and efficacy of tofacitinib in the treatment of AS. Searches were limited to studies published in English between January 01, 2015, and February 01, 2025, to ensure the inclusion of the most recent advancements. The search strategy combined Medical Subject Headings (MeSH) and free-text terms related to “tofacitinib,” “ankylosing spondylitis,” “efficacy,” and “safety.” Reference lists of included articles and relevant reviews were also screened to identify additional studies not captured through the database searches. All identified records were imported into Rayyan, and duplicates were removed. Titles and abstracts of the retrieved records were independently screened by two reviewers to assess relevance. Studies deemed potentially eligible were retrieved in full text for further evaluation. Discrepancies in selection were resolved through discussion and, where necessary, consultation with a third reviewer to achieve consensus. A PRISMA flow diagram was constructed to depict the selection process, including the number of records identified, screened, excluded, and included in the final analysis.

Eligibility Criteria

Studies were included in the review if they met the following criteria: (1) original clinical studies involving human participants; (2) evaluated the efficacy and/or safety of tofacitinib in patients with a clinical diagnosis of AS based on recognized classification criteria such as the modified New York (mNY) criteria or the ASAS criteria; (3) reported at least one clinical efficacy outcome (e.g., ASAS20, ASAS40, BASDAI, or disease activity measures) or safety outcome (e.g., adverse events, serious adverse events, infection rates, or laboratory abnormalities); and (4) randomized controlled trials (RCTs), open-label trials, or prospective observational studies. Exclusion criteria were as follows: (1) case reports, review articles, editorials, and conference abstracts without full data; (2) preclinical studies or studies conducted on animal models; (3) studies involving patients with other forms of spondyloarthritis or inflammatory arthritis where outcomes specific to AS could not be separated; and (4) articles not available in English. Where multiple studies reported on overlapping populations, the most comprehensive or latest report was included to avoid duplication of data.

Data Extraction

Two reviewers independently extracted data from the included studies using a predefined data extraction form. The extracted data included study characteristics (first author, year of publication, country, and study design), participant characteristics (sample size, age, sex distribution, disease duration, and baseline disease activity), intervention details (dose and duration of tofacitinib treatment), comparator (if any), follow-up period, efficacy outcomes (such as ASAS20, ASAS40, and BASDAI scores), and safety outcomes (types and incidence of adverse events, serious adverse events, discontinuation rates, and specific complications such as infections or laboratory abnormalities).

Any discrepancies in data extraction were resolved by consensus or by involving a third reviewer. Where essential data were missing or unclear, attempts were made to contact the corresponding authors of the original studies for clarification. All extracted data were recorded systematically to ensure consistency and transparency in reporting.

Data Analysis

As the available data varied in terms of outcome measures, study designs, and follow-up durations, a meta-analysis was not performed. Instead, a qualitative synthesis of the included studies was conducted. The findings were presented in a narrative format, highlighting patterns in the reported efficacy and safety outcomes of tofacitinib in AS. Key outcomes were summarized study-wise, with attention to consistency in treatment responses and adverse event profiles. Where applicable, variation in treatment effect based on dosage or treatment duration was noted. No statistical pooling or quantitative comparison of effect sizes was undertaken due to the heterogeneity of the included studies.

Results

Study Selection Process

**Figure 1:**
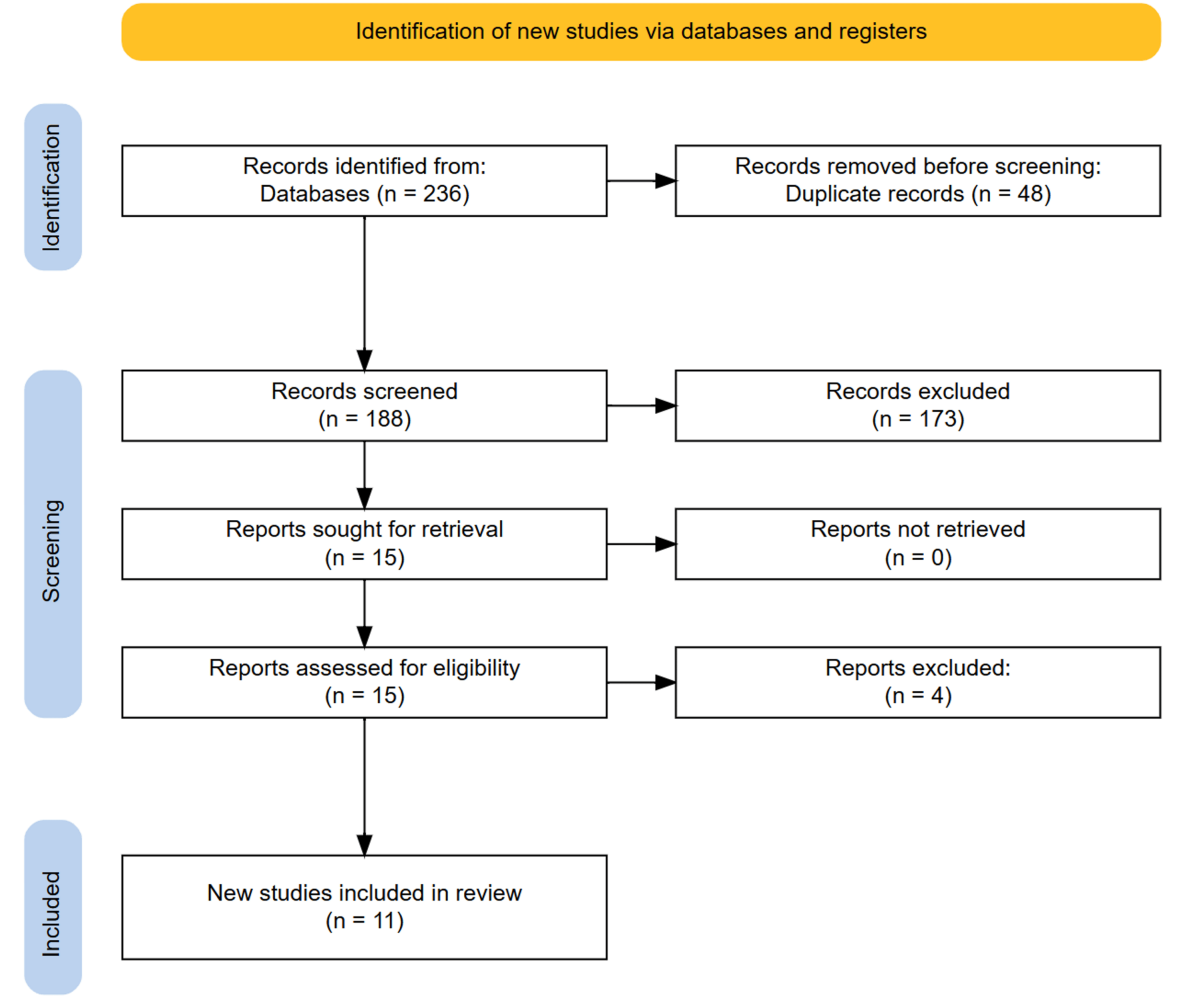
PRISMA diagram illustrating the study selection process PRISMA, Preferred Reporting Items for Systematic Reviews and Meta-Analyses

A total of 236 records were initially retrieved through database searching. Following the removal of 48 duplicates, 188 articles were screened based on their titles and abstracts. Of these, 15 potentially relevant studies were selected for full-text assessment. After detailed evaluation, 11 studies met the eligibility criteria and were included in the final synthesis. No further studies were identified through manual screening of reference lists. The study selection process is illustrated in the PRISMA flow diagram (Figure 1).

Study Characteristics

The literature search yielded multiple relevant studies evaluating tofacitinib in AS. After removing duplicates and screening titles and abstracts, 11 full-text articles met the inclusion criteria. These comprised two RCTs and eight post-hoc or pooled analyses derived from these trials. One real-world retrospective study was also included [9]. Studies excluded during full-text review mainly consisted of conference abstracts without complete data, articles not focusing specifically on AS, or those not evaluating the efficacy or safety of tofacitinib in AS. The final analysis included studies published between 2017 and 2024. Sample sizes varied across studies, ranging from 137 to 372 patients. Most studies utilized the mNY criteria for diagnosing AS, with central reading of sacroiliac joint radiographs for confirmation. One study used the ASAS classification criteria for axial spondyloarthritis [9]. The predominant tofacitinib dosage evaluated was 5 mg twice daily, although some studies also investigated 2 and 10 mg twice-daily regimens. Follow-up periods ranged from 12 to 48 weeks. Efficacy assessments primarily included standard outcome measures such as ASAS20/40 responses, BASDAI scores, ASDAS improvements, and patient-reported outcomes for pain, fatigue, and quality of life. Several studies also evaluated MRI-based inflammation scores using Spondyloarthritis Research Consortium of Canada (SPARCC) or Canada-Denmark (CANDEN) scoring systems [8,10,11]. Safety outcomes typically included treatment-emergent adverse events, serious adverse events, and discontinuation rates due to adverse events, with particular attention to infections, laboratory abnormalities, and cardiovascular events (Table 1).

**Table 1:** Study characteristics and main findings of studies included in this systematic review AS, ankylosing spondylitis; BMI, body mass index; BID, twice daily; RCT, randomized controlled trial; MIC, minimally important change; ASAS, Assessment of Spondyloarthritis International Society; ASDAS, Ankylosing Spondylitis Disease Activity Score; BASDAI, Bath Ankylosing Spondylitis Disease Activity Index; BASMI, Bath Ankylosing Spondylitis Metrology Index; BASFI, Bath Ankylosing Spondylitis Functional Index; CRP, C-reactive protein; mNY, modified New York; FACIT-F, Functional Assessment of Chronic Illness Therapy-Fatigue; TEAEs, treatment-emergent adverse events; SAEs, serious adverse events; AEs, adverse events; IRs, incidence rates; MACE, major adverse cardiovascular events; NMSC, non-melanoma skin cancer; AESI, adverse events of special interest; SPARCC, Spondyloarthritis Research Consortium of Canada; MRI, magnetic resonance imaging; SI, sacroiliac; CANDEN, Canada-Denmark; ASDAS-CRP, ASDAS with CRP; ASDAS-ESR, ASDAS with erythrocyte sedimentation rate; ASDAS MI, major improvement; ASDAS CII, clinically important improvement; ASDAS ID, inactive disease; ASQoL, ankylosing spondylitis quality of life; WPAI, work productivity and activity impairment; GI, gastrointestinal

Author	Year	Study design	Sample size	Diagnostic criteria for AS	Tofacitinib dose	Comparator	Duration of follow-up	Efficacy outcomes	Safety outcomes	SAEs	Conclusions
Ogdie et al. [12]	2024	Post-hoc analysis of pooled data from one phase 2 and one phase 3 RCT	372 patients with AS (178 current/past smokers and 194 never smokers)	Not mentioned	Tofacitinib 5 mg BID (also evaluated 2 mg and 10 mg BID doses in phase 2 trial)	Placebo	48 weeks (efficacy assessed to week 16)	ASAS20, ASAS40, ASDAS < 2.1, BASDAI50, ≥50% improvement in back pain, and FACIT-F≥40.1 rates. Tofacitinib showed greater efficacy vs. placebo across outcomes, with comparable efficacy between current/past and never smokers.	TEAEs, SAEs, and discontinuations due to AEs. TEAE-adjusted IRs were higher in current/past vs. never smokers. SAEs and discontinuation due to AEs were similar between smoking groups.	SAE incidence rates were similar between current/past smokers and never smokers in the tofacitinib groups. No MACE, malignancies excluding NMSC, venous thromboembolism, or other AESI were reported in AS patients.	Tofacitinib efficacy was greater vs. placebo, and comparable across smoking categories in patients with AS. Adjusted IRs were higher in current/past vs. never smokers for TEAEs in AS, complementing reports of associations between smoking and comorbidities in spondyloarthritis. Findings support increased surveillance/caution for patients with AS with a smoking history.
Norton et al. [13]	2025	Post-hoc analysis of pooled data from phase 2/3 RCTs	370 patients (153 with BMI <25 kg/m², 131 with BMI ≥25 to <30 kg/m², and 86 with BMI ≥30 kg/m²)	mNY criteria for AS	Tofacitinib 5 mg BID	Placebo	12 weeks for efficacy outcomes and 16 weeks for safety outcomes	Greater efficacy with tofacitinib vs. placebo across all BMI categories for most outcomes (ASAS20, ASDAS-CRP improvements, BASDAI, BASMI, BASFI, total back pain, nocturnal spinal pain, CRP levels); some exceptions in BMI ≥30 kg/m² category for ASAS40, ASAS partial remission, BASDAI50, and ASDAS-CRP inactive disease rates.	Generally comparable across BMI categories; higher rates of AEs (55.7%) and SAEs (3.8%) in tofacitinib-treated patients with BMI <25 kg/m²; most frequent AEs included nasopharyngitis, upper respiratory tract infection, and arthralgia.	Higher in tofacitinib-treated patients with BMI <25 kg/m² (3.8%) compared to other BMI categories.	Tofacitinib can be considered as a treatment option for patients with active AS regardless of baseline BMI category; interpretation limited by small sample sizes and differences in baseline characteristics across BMI categories.
Navarro-Compán et al. [14]	2024	Post-hoc analysis of a phase 3, RCT (NCT03502616)	269 patients (133 randomized to tofacitinib and 136 randomized to placebo-to-tofacitinib)	mNY criteria for AS (documented with central reading of the radiograph of the sacroiliac joints)	5 mg BID	Placebo (weeks 0-16), then all patients received open-label tofacitinib 5 mg BID until week 48	48 weeks	Median time to improvement in: total back pain, nocturnal pain, BASDAI questions (fatigue, spinal pain, peripheral joint pain/swelling, enthesitis, and morning stiffness), BASDAI total score, and ASDAS.	Not mentioned	Not mentioned	Improvements in AS core domains occurred more rapidly with tofacitinib vs. placebo-to-tofacitinib. Half of the tofacitinib-treated patients experienced improvements ≥30% in pain and ≥1.1 points in ASDAS during month 1, ≥50% improvement in nocturnal pain and enthesitis by month 2, and in morning stiffness by month 3. Initiating tofacitinib as soon as possible was associated with quicker improvements in AS core domains vs. delaying treatment.
Gossec et al. [15]	2025	Post-hoc analysis of a phase 3 RCT	269 patients (133 tofacitinib and 136 placebo)	mNY criteria for AS	5 mg BID	Placebo	16 weeks (double-blind phase of a 48-week trial)	Median time to initial and stable improvements in FACIT-F scores was significantly shorter with tofacitinib vs. placebo. 70.0% vs. 48.5% of patients experienced clinically meaningful initial improvements in FACIT-F total score (≥6 points).	Not mentioned	Not mentioned	Improvements in fatigue occurred more rapidly with tofacitinib than with placebo. Median time to clinically meaningful initial and stable improvements in FACIT-F total score was 8 and 12 weeks, respectively, with tofacitinib but not reached within 16 weeks with placebo. These results may help healthcare providers discuss treatment expectations with patients.
Østergaard et al. [11]	2023	Post-hoc analysis of a 16-week, phase 2, multicenter, randomized, double-blind, placebo-controlled, dose-ranging trial (NCT01786668)	137 patients (tofacitinib 5 mg BID: n = 46; tofacitinib 10 mg BID: n = 47; placebo: n = 44)	mNY criteria for AS	Tofacitinib 5 and 10 mg BID (the 2 mg BID group was excluded from this analysis as previously found not efficacious)	Placebo	12 weeks (for MRI assessments)	Significant reductions in CANDEN spine inflammation scores: total spine inflammation score (p < 0.0001), vertebral body inflammation (p < 0.0001), posterior elements inflammation (p < 0.0001), corner inflammation (p < 0.0001), non-corner inflammation (p < 0.05), facet joint inflammation (p < 0.0001), and posterolateral inflammation (p < 0.0001) subscores vs. placebo.	Not mentioned	Not mentioned	In patients with AS, tofacitinib treatment was associated with significant reductions in MRI scores of spinal inflammation vs. placebo, as assessed by the CANDEN MRI scoring system. Tofacitinib reduced inflammation in the posterolateral elements of the spine and facet joints, which has not been described previously.
Kristensen et al. [16]	2023	Pooled analysis of a 16-week phase 2 randomized, double-blind, placebo-controlled trial (NCT01786668) and a 48-week phase 3 randomized, double-blind, placebo-controlled trial (NCT03502616)	370/371 patients included in models A/B	Not mentioned	5 mg BID	Placebo	Phase 2: 16 weeks (12-week treatment + 4-week washout); phase 3: 48 weeks (16-week double-blind phase followed by open-label tofacitinib to week 48)	Improvement in fatigue measured by FACIT-F and BASDAI Q1; improvements in pain (back pain, nocturnal spinal pain, and BASDAI Q2/3); improvements in morning stiffness (BASDAI Q5/6); reductions in CRP.	Not mentioned	Not mentioned	In tofacitinib-treated patients with AS, improvements in fatigue were collectively mediated through combined treatment effects on morning stiffness and pain. The analysis showed that tofacitinib treatment affects fatigue mainly indirectly via pain and morning stiffness pathways rather than through direct effects or via CRP.
Ganapati et al. [9]	2023	Retrospective study of prospectively collected data from nine rheumatology centers	168	The ASAS classification criteria for axSpA	5 mg BID	No comparator (real-world clinical setting with patients on tofacitinib generics)	6 months	At 6 months: 57.9% achieved CII; median decrease in ASDAS ESR score was 2.02 (1.18, 2.96); 50.6% achieved major improvement in ASDAS ESR.	Most common adverse events: weight gain (n = 8), urinary tract infection (n = 2), upper respiratory infection (n = 2), herpes zoster (n = 2); no cases of tuberculosis, deep vein thrombosis, or adverse cardiovascular events.	One case of lobar pneumonia required the temporary discontinuation of tofacitinib.	This real-world study provides evidence supporting the csDMARD and NSAID-sparing ability of tofacitinib generics in the treatment of axSpA. Tofacitinib generics displayed a good safety profile and showed signals of efficacy as well.
Navarro-Compán et al. [6]	2022	RCT	269 patients (133 tofacitinib and 136 placebo→tofacitinib)	mNY criteria with central reading of sacroiliac joint radiographs, BASDAI score ≥4, and back pain score ≥4	5 mg BID	Placebo for 16 weeks, then all patients received open-label tofacitinib	48 weeks	Greater improvements with tofacitinib vs. placebo at week 16 in pain (BASDAI overall spinal pain: −2.85 vs. −1.34), fatigue (BASDAI fatigue: −2.36 vs. −1.08), quality of life (ASQoL: −4.03 vs. −2.01), and work productivity (WPAI overall work impairment: −21.49 vs. −7.64); all p < 0.001. Improvements continued or increased to week 48. Patients reported clinically meaningful PRO improvements at week 16.	Not mentioned	Not mentioned	In patients with AS, treatment with tofacitinib 5 mg twice daily resulted in clinically meaningful improvements in pain, fatigue, HRQoL, and work productivity vs. placebo to week 16, which were sustained to week 48.
Ogdie et al. [7]	2020	Post-hoc analysis of 7 double-blind, placebo-controlled RCTs	For the AS population: not explicitly stated in the extract, but was part of a larger analysis including 3330 patients across 7 studies	mNY criteria for AS (confirmed by centralized reading of sacroiliac radiographs) and active disease based on BASDAI score ≥4 and back pain score (BASDAI Q2) ≥4	5 and 10 mg BID	Placebo	12 weeks for the AS population	In AS population: significant improvements in SF-36v2 BP domain scores at week 12 with both tofacitinib doses vs. placebo (p < 0.01); improvements in BASDAI Q2 (neck, back, and hip pain) and Q3 (peripheral pain/swelling); reduced percentage of patients answering "yes" to ASQoL Q9 and Q14; improvement in EQ-5D pain/discomfort dimension (significant for 10 mg BID but not 5 mg BID).	Not mentioned	Not mentioned	Tofacitinib was associated with rapid and sustained improvements across multiple pain measures in patients with AS (and other inflammatory rheumatic musculoskeletal diseases).
Maksymowych et al. [10]	2018	post hoc analysis of a phase II, multicentre, randomized, double-blind, placebo-controlled, dose-ranging study (NCT01786668)	207 patients (164 with MRI data at baseline and follow-up)	mNY criteria for AS, confirmed by centralized reading of sacroiliac radiographs	Tofacitinib 2, 5, and 10 mg BID	Placebo	16 weeks (12-week treatment + 4-week washout/off-treatment follow-up period)	Primary: SPARCC MRI scores for SI joint and spine inflammation. Significantly greater improvements from baseline in SPARCC SI joint and spine scores with tofacitinib vs. placebo. Greater proportions of patients achieved minimally important change in SPARCC SI joint scores with tofacitinib (2 mg: 28.6%, 5 mg: 38.6%, and 10 mg: 29.6%) vs. placebo (11.8%). Similarly, for spine scores with tofacitinib (2 mg: 29.3%, 5 mg: 36.4%, and 10 mg: 40.9%) vs. placebo (11.8%). Secondary: clinical endpoints including ASAS20, ASAS40, ASDAS MI, ASDAS CII, and ASDAS ID, and changes in BASDAI, BASFI, and total back pain.	Not mentioned	Not mentioned	Approximately one-third of the tofacitinib-treated AS patients experienced clinically meaningful reductions in spinal MRI inflammation at week 12. Patients achieving a MIC for MRI inflammation had a greater clinical response.
van der Heijde et al. [8]	2017	RCT	208 randomized, 207 treated patients (51 placebo and 52 in each tofacitinib group)	mNY criteria for AS, confirmed by centralized reading of sacroiliac radiographs	Three doses: 2, 5, or 10 mg BID	Placebo	16 weeks (12-week treatment, 4-week washout)	Primary: ASAS20 response at week 12. Secondary: ASAS40, ASAS5/6, ASDAS improvements, BASDAI50, SPARCC SI joint and spine scores, and Berlin score. Tofacitinib 5 mg twice daily showed significantly higher ASAS20 responses vs. placebo (80.8% vs. 41.2%; p < 0.001). All tofacitinib doses showed significant improvement in ASAS40, BASDAI50, and ASDAS vs. placebo.	Generally similar AE rates across treatment groups. Most common TEAEs: nasopharyngitis (n = 13) and upper respiratory tract infection (n = 8). Dose-dependent laboratory changes returned to baseline after a 4-week washout.	One serious infection (chronic iridocyclitis/uveitis) in the tofacitinib 5 mg group was considered unrelated to treatment. Two treatment-related herpes zoster cases (one each in the tofacitinib 2 and 10 mg groups). No tuberculosis, malignancies, GI perforations, or deaths reported.	Tofacitinib 5 and 10 mg BID demonstrated greater clinical efficacy vs. placebo in reducing signs, symptoms, and spinal inflammation of AS. The 12-week safety profile was similar to tofacitinib studies in other indications, with no new safety signals. JAK inhibition may present a new mode of action for managing AS.

Discussion

This systematic review synthesized evidence from 11 studies evaluating the efficacy and safety of tofacitinib in the treatment of AS. The findings collectively suggest that tofacitinib demonstrates significant clinical benefits across multiple domains of disease activity, with an acceptable safety profile consistent with its known risks in other inflammatory conditions.

The efficacy of tofacitinib in AS is supported by consistent improvements in standard disease activity measures across all included studies. Two pivotal RCTs demonstrated statistically significant and clinically meaningful improvements in ASAS20/40 response rates, BASDAI scores, and ASDAS metrics compared to placebo [6,8]. In the phase 2 trial by van der Heijde et al., tofacitinib 5 mg BID yielded ASAS20 response rates of 80.8% compared to 41.2% with placebo (p < 0.001), establishing the clinical efficacy of this dosage [8]. These findings were corroborated in the phase 3 trial, with sustained benefits observed through 48 weeks of follow-up. Importantly, improvements were demonstrated across diverse patient subgroups, including those stratified by BMI categories and smoking status, suggesting that tofacitinib may be effective regardless of these clinical characteristics [12,13].

Beyond conventional clinical endpoints, several studies investigated the effects of tofacitinib on specific symptom domains highly relevant to patient experience. Notably, improvements in pain measures were rapid and substantial, as demonstrated by Navarro-Compán et al., who reported that half of the tofacitinib-treated patients experienced ≥30% improvements in pain within the first month of treatment [14]. Similarly, fatigue, a pervasive and debilitating symptom in AS, showed significant improvement with tofacitinib compared to placebo, with median time to clinically meaningful improvement in Functional Assessment of Chronic Illness Therapy-Fatigue (FACIT-F) scores being substantially shorter in the tofacitinib group [15]. The mediation analysis by Kristensen et al. provided mechanistic insights, suggesting that the effects of tofacitinib on fatigue are primarily mediated through improvements in pain and morning stiffness rather than through direct mechanisms or reductions in systemic inflammation (CRP) [16]. A particularly compelling aspect of the efficacy profile of tofacitinib is its demonstrated impact on objective measures of disease activity. Three studies evaluated the effect of tofacitinib on MRI-detected inflammation using validated scoring systems [8,10,11]. Maksymowych et al. reported that approximately one-third of the tofacitinib-treated patients achieved minimally important changes (MICs) in SPARCC scores for both sacroiliac joint and spinal inflammation at week 12 [10]. Østergaard et al. further characterized these effects using the CANDEN MRI scoring system, demonstrating significant reductions in multiple components of spinal inflammation, including vertebral body inflammation, posterior elements, facet joints, and corner inflammation [11]. These findings align with the known mechanism of action of JAK inhibition, which targets multiple cytokine pathways implicated in spondyloarthritis pathogenesis. Patient-reported outcomes reflecting health-related quality of life and functional capacity also improved significantly with tofacitinib treatment. Navarro-Compán et al. reported substantial improvements in ankylosing spondylitis quality of life (ASQoL) scores and work productivity and activity impairment (WPAI), which were both statistically significant compared to placebo and exceeded thresholds for clinical meaningfulness [6]. These benefits are particularly important given the substantial impact of AS on patients’ daily functioning and socioeconomic outcomes.

These review findings indicate that the safety profile of tofacitinib in AS is generally consistent with observations in other inflammatory diseases. Across studies, treatment-emergent adverse events were mainly mild to moderate, with nasopharyngitis and upper respiratory tract infections being most commonly reported [8,13]. Serious adverse events were infrequent, although some studies noted a numerically higher incidence in certain subgroups, such as patients with a BMI <25 kg/m² [13]. The two cases of herpes zoster reported by van der Heijde et al. are consistent with the known risk of this infection with JAK inhibition [8]. Notably, no cases of tuberculosis, malignancies, gastrointestinal perforations, major adverse cardiovascular events, or venous thromboembolism were reported within the study periods, though these remain established risks with JAK inhibitor therapy that require monitoring. The real-world evidence provided by Ganapati et al. complements the controlled trial data, demonstrating the effectiveness and safety of tofacitinib generics in clinical practice [9]. Their report, which states that 57.9% of patients achieved clinically important improvement and 50.6% achieved major improvement in ASDAS-ESR scores after six months, supports the external validity of the RCT findings. Furthermore, this study highlighted the steroid-sparing effect of tofacitinib, a valuable attribute for the long-term management of chronic inflammatory diseases.

Interestingly, post-hoc analyses revealed potential predictors of response and timing of benefit for specific AS domains. Norton et al. suggested that efficacy may be somewhat attenuated in patients with a BMI ≥30 kg/m² for certain endpoints, although tofacitinib remained effective across all BMI categories [13]. The time-to-event analyses conducted by Navarro-Compán et al. and Gossec et al. provide clinically useful information about the expected timing of improvements across various disease domains, with pain and ASDAS improvements occurring most rapidly (within one month), while improvements in morning stiffness and fatigue followed a more gradual course [14,15].

Limitations and future directions

This systematic review has several limitations that warrant consideration. First, the evidence base for tofacitinib in AS remains relatively limited, with only two original RCTs identified, supplemented by multiple post-hoc analyses. Second, the maximum follow-up duration was 48 weeks, precluding assessment of long-term efficacy and safety outcomes, which are particularly relevant for a chronic condition like AS. Third, most studies included predominantly biologic-naïve patients, limiting conclusions about the efficacy of tofacitinib in patients with inadequate response to biologics. Fourth, comparisons with active treatments, particularly TNF inhibitors or IL-17 antagonists, were absent, making it impossible to determine the relative efficacy of tofacitinib compared to established therapies. Finally, the heterogeneity in outcome measures and reporting precluded formal meta-analysis, limiting the precision of efficacy estimates.

Future research should address these gaps by conducting head-to-head trials comparing tofacitinib with biologic DMARDs, evaluating longer-term outcomes beyond one year, and investigating predictive biomarkers of response. Studies examining the efficacy of tofacitinib specifically in biologic-experienced patients would inform its positioning in treatment algorithms. Additionally, real-world effectiveness studies with larger patient cohorts would complement the controlled trial data and better characterize the risk of uncommon adverse events. Finally, investigation of combination therapies, dose optimization strategies, and the potential role of tofacitinib in preventing radiographic progression would further define its place in AS management.

## Conclusions

This systematic review provides comprehensive evidence supporting the efficacy and safety of tofacitinib in the treatment of AS. Across multiple studies, tofacitinib 5 mg twice daily consistently demonstrated significant improvements in clinical symptoms, disease activity measures, patient-reported outcomes, and objective inflammatory markers compared to placebo. These benefits were observed across diverse patient subgroups and were maintained with continued treatment. Particularly noteworthy were the rapid onset of action for pain relief, substantial improvements in fatigue, and significant reductions in MRI-detected spinal inflammation. The oral administration route represents a practical advantage over injectable biologics for many patients. Safety findings were consistent with the established profile of tofacitinib in other inflammatory conditions, with no new safety signals identified specific to AS. While more common adverse events included nasopharyngitis and upper respiratory infections, serious adverse events were infrequent. However, the known risks associated with JAK inhibition, including herpes zoster reactivation and potential cardiovascular events, necessitate appropriate patient selection and monitoring. Given the limited therapeutic options for patients with AS who fail or cannot tolerate first-line treatments, tofacitinib emerges as a valuable addition to the treatment armamentarium. Future research should focus on long-term outcomes, comparative effectiveness against established biologics, and identification of predictive response biomarkers to optimize treatment selection for individual patients.
